# Thermal strain is greater in the late afternoon than morning during exercise in the gym without airflow and air conditioning on a clear summer day

**DOI:** 10.3389/fspor.2023.1147845

**Published:** 2023-02-28

**Authors:** Hidenori Otani, Takayuki Goto, Yuki Kobayashi, Heita Goto, Minayuki Shirato, Yuri Hosokawa, Ken Tokizawa, Mitsuharu Kaya

**Affiliations:** ^1^ Faculty of Health Care Sciences, Himeji Dokkyo University, Himeji, Japan; ^2^ National Institute of Technology, Akashi College, Akashi, Japan; ^3^ Kyushu Kyoritsu University, Kitakyushu, Japan; ^4^ Meiji Gakuin University, Tokyo, Japan; ^5^ Faculty of Sport Sciences, Waseda University, Tokorozawa, Japan; ^6^ National Institute of Occupational Safety and Health, Kiyose, Japan; ^7^ School of Rehabilitation, Hyogo Medical University, Kobe, Japan

**Keywords:** body temperature, heat-related illness, heat stress, indoor sports, time-of-day

## Abstract

**Introduction:**

There are no reports examining the time-of-day effect on team training sessions in the gym without airflow and air conditioning on thermal strain in the summer heat. We investigated this effect during badminton training sessions on a clear summer day.

**Methods:**

Nine male high school badminton players (Mean ± SD; age 17.1 ± 0.6 y, height 171 ± 4 cm, body mass 59 ± 7 kg) completed two 2.5-h badminton training sessions in the gym without airflow and air conditioning. The training sessions were started at 0900 h (AM) and 1600 h (PM) on separate days in August. Skin temperatures (chest, triceps, thigh, calf), infrared tympanic temperature, heart rate, thermal sensation and rating of perceived exertion were recorded at rest and at regular intervals during the sessions.

**Results:**

Indoor and outdoor environmental heat stress progressively increased in AM and decreased in PM during the sessions. Ambient temperature (AM 30.1 ± 0.9°C; PM 33.2 ± 1.0°C: *P *< 0.001) and wet-bulb globe temperature (AM 28.1 ± 0.5°C; PM 30.0 ± 0.9°C: *P *= 0.001) during the sessions in the gym were higher in PM than AM. Mean skin temperature (AM 34.2 ± 1.0°C; PM 34.7 ± 0.7°C: *P *< 0.001), infrared tympanic temperature (AM 37.8 ± 0.5°C; PM 38.1 ± 0.4°C: *P *= 0.001) and thermal sensation (AM 2.7 ± 1.4; PM 3.3 ± 1.0: *P *< 0.001) during the sessions were higher in PM than AM. Body heat storage (AM 159 ± 30 W·m^−2^; PM 193 ± 30 W·m^−2^: *P *< 0.05) was greater in PM than AM. There were no time-of-day differences in the average heart rate (AM 75 ± 4% age-predicted maximal heart rate; PM 76 ± 5 age-predicted maximal heart rate: *P *= 0.534), body mass loss (AM 0.6 ± 0.3 kg; PM 0.8 ± 0.2°C: *P *= 0.079), the volume of water ingested (AM 1.5 ± 0.1 L; PM 1.6 ± 0.3 L: *P *= 0.447) and rating of perceived exertion (AM 16 ± 2; PM 16 ± 3: *P *= 0.281).

**Conclusions:**

This study indicates greater thermal strain in PM trial than in AM trial during team training sessions in the gym without airflow and air conditioning on a clear summer day. Therefore, athletes and coaches of indoor sports should perceive that athletes may be exposed to a greater risk for thermal strain in the late afternoon from 1600 h than in the morning from 0900 h during the sessions in the gym under these conditions.

## Introduction

The greater thermal strain and risk of exertional heat-related illness have been reported in the morning from 0900 h (AM) than in the late afternoon from 1600 h (PM) in high school athletes during outdoor (1, 2) and indoor (3) team training sessions in hot summer environmental conditions under a clear sky. During 3-h baseball (1) and 2-h soccer (2) training sessions, there were higher mean skin temperature (T_sk_), infrared tympanic temperature (T_ty_) and heart rate (HR) in AM than PM trial when no time-of-day differences in ambient temperature (T_a_) and wet-bulb globe temperature (WBGT) were existed. Moreover, during 2.5-h judo training sessions, there was high T_sk_ in AM than PM trial when higher T_a_ in PM than AM trial with no time-of-day difference in WBGT was observed (3). These studies therefore concluded a greater thermal strain in AM than PM trial because of an increase in environmental heat stress with increasing solar radiation during AM trial compared with a decrease in environmental heat stress with decreasing solar radiation during PM trial (1, 2, 3). However, since only one study (3) has investigated the time-of-day effect of indoor team training sessions on the risks of thermal strain during exercise, further investigation is warranted.

While many indoor sports are conducted in the gym even in the summer heat, if there is limited airflow and no air conditioning, the risks of thermal strain and exertional heat-related illness may increase (4, 5). This is because airflow during exercise-heat stress can attenuate increases in core temperature (T_c_), T_sk_ and HR and elevate evaporative heat loss (EHL) with increasing air velocity (6–9). However, high airflow will disturb performance in some indoor sports such as badminton and table tennis. For example, airflow must be restricted in badminton and table tennis to prevent the wind from affecting the shuttlecock and ball flight (10, 11). Athletes of these sports may be therefore exposed to greater heat stress in the summer. In fact, badminton reported the highest number of exertional heat-related illness in Japanese high school indoor-sports athletes (12). The badminton training sessions have also been known to be involved with a lot of intermittent exercise, which could potentially result in greater thermal strain as the metabolic heat production, (M–W) cannot be successfully dissipated into the environment due to limited airflow (13). That said, elevated body temperature does not only have negative effects. It is demonstrated that a 1°C increase in muscle temperature can improve muscle performance during short duration exercise (14). Nevertheless, the diurnal impacts of the badminton training sessions in the gym without airflow and air conditioning in hot environmental conditions on thermal strain in high school athletes have not been examined to our knowledge.

Therefore, the aim of the present study was to examine the time-of-day influence of the badminton training sessions in the gym without airflow and air conditioning on thermal strain in high school athletes in the summer heat. We hypothesised that the thermal strain would be greater in AM than PM trial owing to an increase in indoor environmental heat stress during AM trial, given the results of previous studies (1–3).

## Material and methods

### Participants

After approval by the University’s Ethics Committee (REF: 21-02), nine healthy males, who were a member of a high school badminton team [mean ± standard deviation (SD); age 17.1 ± 0.6 year, height 171 ± 4 cm, body mass 59 ± 7 kg, body mass index 20 ± 2 kg·m^−2^], completed the study. They completed a similar protocol of training in this study in the gym, which had no air-conditioning, for more than 18 weeks (6 days·week^−1^). The experiments were conducted in August to ensure that participants were naturally acclimated to the heat. Participants and their parents received written information regarding the aim and nature of this study and provided written consent before participation.

### Experimental design

Participants performed two 2.5-h regular badminton training sessions in a gym. The gym was a one-story building where the floor space was 800 m^2^ (40 m  ×  20 m) and the air volume was 8,000 m^3^ (40 m  ×  20 m  ×  10 m). There were east and west facing windows which were kept close during the sessions. The sessions were started at two different times-of-day: 0900 h (AM trial) and 1600 h (PM trial). The sessions were conducted on a completely clear day at PM trial first in early-August and at AM trial performing 22 days later in late-August. Between the sessions, weekly daily mean ambient temperature in the experimental area was stable and about 28.0°C during both early (PM trial) and late (AM trial) August. A normal training session was completed 2 days before the trials but participants were asked not to perform any strenuous exercise in the 24 h before the experimental trials. Between PM and AM trials, participants trained 6 days·week^−1^ performing a similar protocol of training in the trials. Participants were clothed in badminton uniform (short-sleeve, crew neck T-shirt; loose-fitting shorts; ankle-length socks; athletic shoes) in both trials. This ensemble was the same as the casual running ensemble and was assumed to be 1.328 kg of total weight, 1.12 of the clothing area factor, 0.063 (m^2^·°C)·W^−1^ or 0.405 clo of the intrinsic clothing insulation and 0.01 (m^2^·kPa)·W^−1^ of the evaporative resistance of clothing (15).

### Experimental protocol

Participants reported to the laboratory after a 3-h fast. They were instructed to drink 500 ml of water until 60 min prior to the start of the trial. Once participants arrived at the laboratory, they first emptied their bladder to measure urine specific gravity (D04–650–0, AS ONE Co., Ltd., Osaka, Japan) and thereafter nude body mass was determined to the nearest 10 g (AD6205B, A&D Co., Ltd., Tokyo, Japan). Euhydration was confirmed upon arrival by a urine specific gravity of ≤1.025. Thermistor probes (ITP082-25, Nikkiso-Therm Co., Ltd., Musashino, Tokyo, Japan) were attached to the skin surface at four locations (chest, triceps, thigh and calf) to determine weighted mean skin temperature (T_sk_ 16):, and a HR telemetry band (H10 transmitter, Polar Electro Oy, Kempele, Finland) was positioned. Gastrointestinal thermometry is a well-accepted device for T_c_ assessment in the athletics and field settings (17). However, this study employed T_ty_ to estimate T_c_ because using gastrointestinal thermometry is restricted by pharmaceutical affairs law in Japan. An infrared tympanic thermometer (GeniusTM 2, Covidien, Mansfield, MA, USA) was used to measure T_ty_. A single operator completed all T_ty_ measurements. Each T_ty_ measurement was obtained two consecutive readings, using the recommended technique (18, 19). A 9-point scale (20) was used to assess thermal sensation (TS). Pre-exercise measurements were conducted in the laboratory in a warm environment (28–29°C).

**Figure 1:**
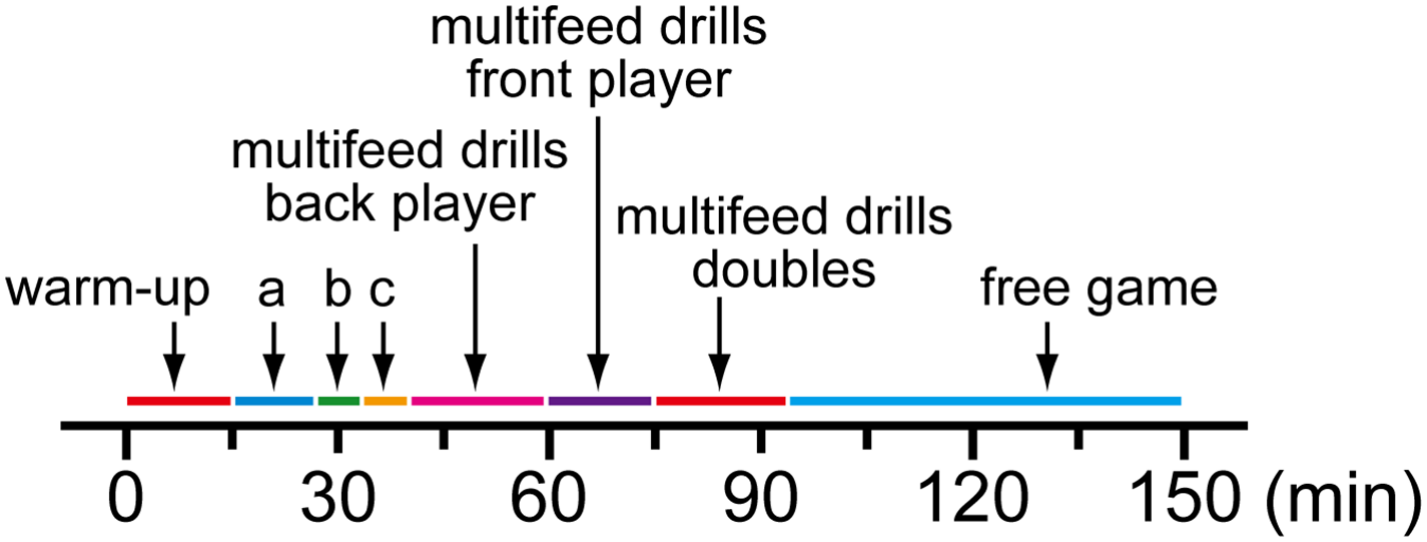
Schematic representation of the experimental protocol. (**a**) Lob shot; (**b**) Smash and net shot; (**c**) Drive and smash.

Participants commenced a 2.5-h training session in a dry badminton uniform and a 4-h fasted state. Participants could freely consume plain water maintained at about 30°C during the sessions. The training session comprised 15 min of warm-up, 11 min of lob shot, 6 min of smash and net shot, 6 min of drive and smash, 22 min of multifeed drills, back player, 15 min of multifeed drills, front player, 20 min of multifeed drills, doubles, 55 min of free game (Figure 1). During the sessions, HR (Polar Team, Polar Electro Oy, Kempele, Finland) was recorded every 15 min and T_ty_, skin temperatures (N543R; Nikkiso-Therm Co., Ltd.) and TS were recorded at 60, 120 and 150 min. At 60, 120 and 150 min, rating of perceived exertion (RPE) was assessed using the 6–20 RPE scale (21) to determine whole-body perception of effort. Following the sessions, participants measured nude body mass after removing the probes.

### Environmental measurements

Indoor environmental condition was determined 1.5 m above the floor in the gym and outdoor environmental condition was assessed 1.5 m above a dark asphalt pavement close to the gym. A WBGT meter (WBGT-203A; Kyoto Electronics Industry Co., Ltd., Fukuchiyama, Kyoto, Japan) was used every 30 min to evaluate T_a_, relative humidity (RH), black globe temperature (T_g_) and WBGT. Floor surface temperature was measured using an infrared thermometer (ISK8700II; AS ONE Corp., Osaka, Japan). A pyranometer (MS-01; Eko Instruments Co., Ltd., Tokyo, Japan) was employed every 30 min to assess direct and diffuse solar radiation in the horizontal plane. Solar radiation (global) was obtained as a sum of the direct and diffuse solar radiation.

### Calculations

Calculations of T_sk_, total sweat loss, age-predicted maximal HR (HRmax), body heat storage (S), M–W, metabolic rate M, heat exchange by radiation (R) and by convection (C), heat loss by evaporation from the skin (E_sk_), respiratory heat loss by convection and evaporation (C_res _+ E_res_) and absolute humidity are included in Supplementary Material (supplementary material).

### Statistical analyses

Statistical significance was accepted at *P* < 0.05. The normality of the data was checked using Shapiro-Wilk’s test. The homogeneity of variance was tested using Levene’s test. The R (version 4.0.2) was used for analysing non-parametric data (TS). A repeated measures two-way (time-of-day [two levels, i.e., AM and PM]  ×  time [four levels, i.e., 0, 60, 120 and 150 min]) ANOVA was conducted on TS with the R package nparLD (version 2.1) for the LD-F2 design (22). Where a significant interaction was apparent, pair-wise differences were evaluated using the Tukey multiple comparison tests. The IBM SPSS (version 21; IBM Corp., Armonk, N.Y., USA) was used for analysing parametric data. A repeated measures two-way (time-of-day [two levels, i.e., AM and PM]  ×  time [four to eleven levels, i.e., 0 to 150 min]) ANOVA was performed on data obtained over time, and paired sample t-tests were conducted on single time point data. Pair-wise differences were evaluated using the Tukey multiple comparison test. The independent samples t-test was performed on environmental parameters (AM vs. PM). Cohen’s d (*d*) was employed as effect size measure; a *d* of 0.2 to <0.5, ≥0.5 to <0.8 and ≥0.8 represents a small, medium and large effect sizes, respectively (23). Data are expressed as mean ± SD.

## Results

Pre-exercise body mass (AM 58.4 ± 6.6 kg, PM 58.1 ± 6.2 kg; *P *= 0.515; *d *= 0.04) and urine specific gravity (AM 1.020 ± 0.002, PM 1.021 ± 0.002; *P *= 0.908; *d *= 0.50) were not different between trials*,* but pre-exercise T_sk_ (*d *= 2.33), T_ty_ (*d *= 2.16), HR (*d *= 2.28) and TS (*d *= 1.12) were higher (all *P *< 0.05) on PM than AM trial (Figures 3, 4).

### Environmental conditions

**Figure 2:**
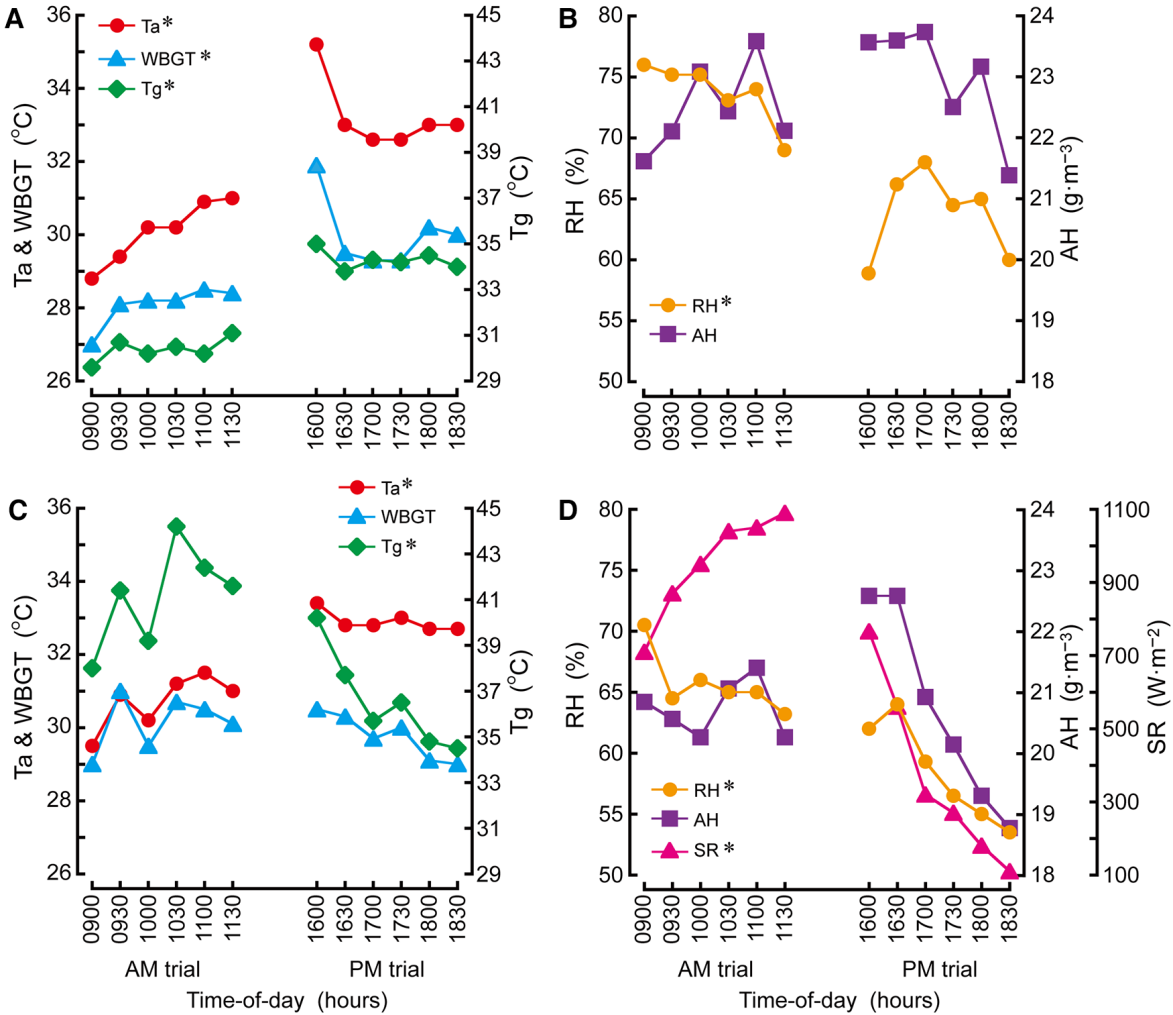
Time-of-day differences in indoor (**A**,**B**) and outdoor (**C**,**D**) environmental conditions. T_a_, ambient temperature; WBGT, wet-bulb globe temperature; T_g_, black globe temperature; RH, relative humidity; AH, absolute humidity; SR, solar radiation. *P < 0.05 denotes a difference between AM and PM trials.

In indoor environmental conditions, T_a_ (*d *= 3.64), T_g_ (*d *= 3.48) and WBGT (*d *= 2.61) were lower (all *P *< 0.05) and RH was higher (*P *< 0.05; *d *= 3.22) on AM than PM trial (Figures 2A,B). In outdoor environmental conditions, T_a_ was lower (*P *< 0.05; *d *= 4.27) and T_g_ (*d *= 2.31), RH (*d *= 2.37) and solar radiation (*d *= 3.16) were higher (all *P *< 0.05) on AM than PM trial (Figures 2C,D).

### Body fluid balance

There were no differences (all *P *≥ 0.05) between trials in body mass loss (*d *= 0.79), the dehydration level (*d *= 0.79), total sweat loss (*d *= 1.00) and the volume of water ingested (*d *= 0.45) (Table 1).

**Table 1:** Body fluid balance during the sessions.

	BML (kg)	Dehydration (%)	TSL (kg)	Rehydration (L)
AM	0.6 ± 0.3	1.0 ± 0.5	2.1 ± 0.3	1.5 ± 0.1
PM	0.8 ± 0.2	1.3 ± 0.2	2.4 ± 0.3	1.6 ± 0.3
*P* value	0.079	0.071	0.065	0.447

Values are mean ± SD. Abbreviations: BML, body mass loss; Dehydration, the dehydration level; TSL, total sweat loss; Rehydration, the volume of water ingested.

### Body temperature responses

**Figure 3:**
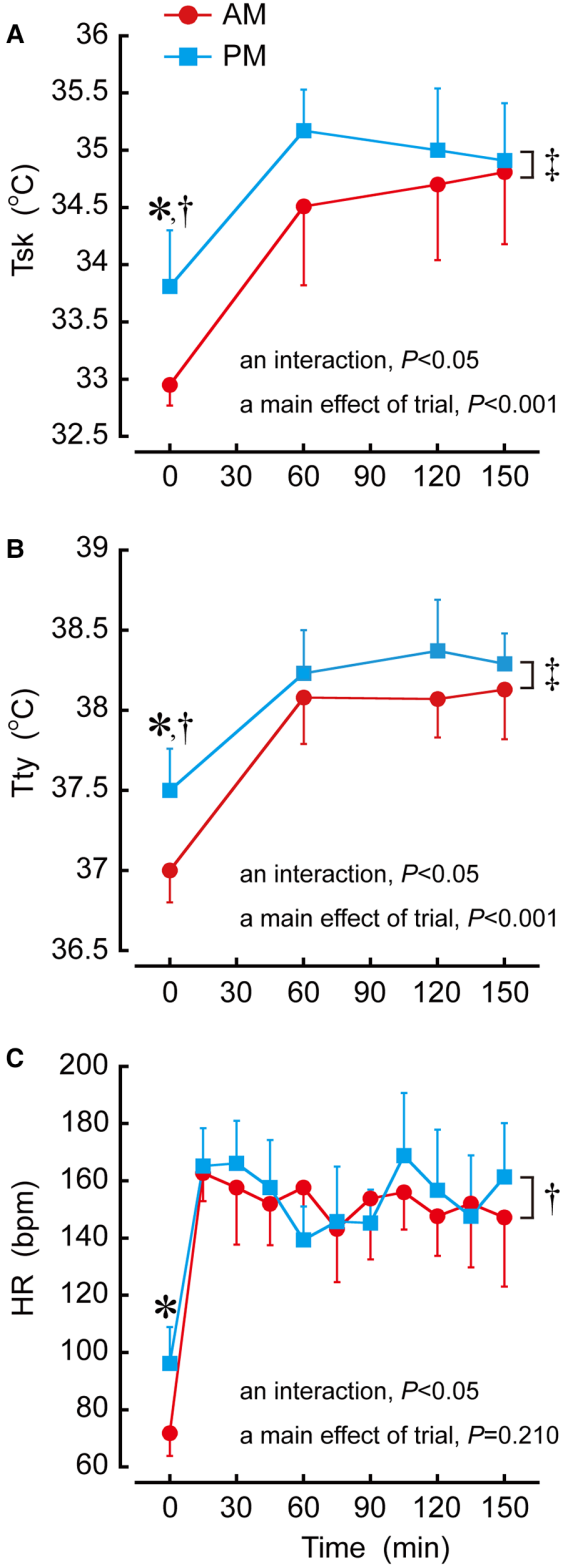
Time-of-day differences in mean skin temperature (**A**: Tsk), infrared tympanic temperature (**B**: Tty) and heart rate (**C**: HR) during exercise. *P < 0.05 denotes a difference of pre-exercise between AM and PM trials. †*P *< 0.05 denotes a trial by time interaction between AM and PM trials. ‡*P *< 0.05 denotes a main effect of trial between AM and PM trials.

There was a trial by time interaction in T_sk_ (*P *< 0.05; Figure 3A) which was higher in PM than AM trial at 0 min (*P *< 0.05; *d *= 2.17). A main effect of trial during the sessions was observed for T_sk_ (*P *< 0.001) which was higher in PM than AM trial (*P *< 0.001; *d *= 0.64). Without 0 min, there was a main effect of trial in T_sk_ (*P *< 0.05) which was higher in PM than AM trial (*P *< 0.05; *d *= 0.66).

A trial by time interaction was shown for T_ty_ (*P *< 0.05; Figure 3B) which was higher in PM than AM trial at 0 min (*P *< 0.05; *d *= 2.02). There was a main effect of trial during the sessions in T_ty_ (*P *< 0.001) which was higher in PM than AM trial (*P *< 0.001; *d *= 0.57). Without 0 min, a main effect of trial was detected for T_ty_ (*P *< 0.05) which was higher in PM than AM trial (*P *< 0.05; *d *= 0.76).

### Heart rate response

A trial by time interaction was shown for HR (*P *< 0.05; Figure 3C), but *post hoc* analysis revealed no difference at the same time point between trials. The average HR during exercise was not different between AM trial (75 ± 4% HRmax) and PM trial (76 ± 5% HRmax: *P *= 0.534).

### Heat balance responses

During the sessions, S (*d *= 1.14) was greater and C_res _+ E_res_ (*d *= 0.91) was less (both *P *< 0.05) on PM than AM trial (Table 2). Also, R and C were negative values in both trials and greater on PM than AM trial, indicating that both heat loss by radiation (R: *d *= 2.50) and by convection (C: *d *= 5.07) were less (both *P* < 0.001) on PM than AM trial (Table 2).

**Table 2:** Heat balance during the sessions.

	S (W · m^−2^)	M–W (W · m^−2^)	M (W · m^−2^)	R (W · m^−2^)	C (W · m^−2^)	E_sk_ (W · m^−2^)	C_res _+ E_res_ (W · m^−2^)
AM	159 ± 30	287 ± 30	312 ± 33	−15 ± 2	−33 ± 5	63 ± 6	16 ± 2
PM	193 ± 30	295 ± 33	320 ± 36	−11 ± 1	−13 ± 3	63 ± 4	15 ± 2
*P* value	0.020	0.529	0.516	<0.001	<0.001	0.938	0.032

Values are mean ± SD. All variables presented as an average of the final 30 min of exercise. Abbreviations: S, heat storage; M–W, metabolic heat production; M, metabolic rate; R, heat exchange by radiation; C, heat exchange by convection; E_sk_, heat loss by evaporation from the skin; C_res _+ E_res_, respiratory heat loss by convection and evaporation. With R and C, positive (+) and negative (−) values are heat gain and loss, respectively. All calculations are included in supporting information (supplementary material).

### Perceptual responses

**Figure 4:**
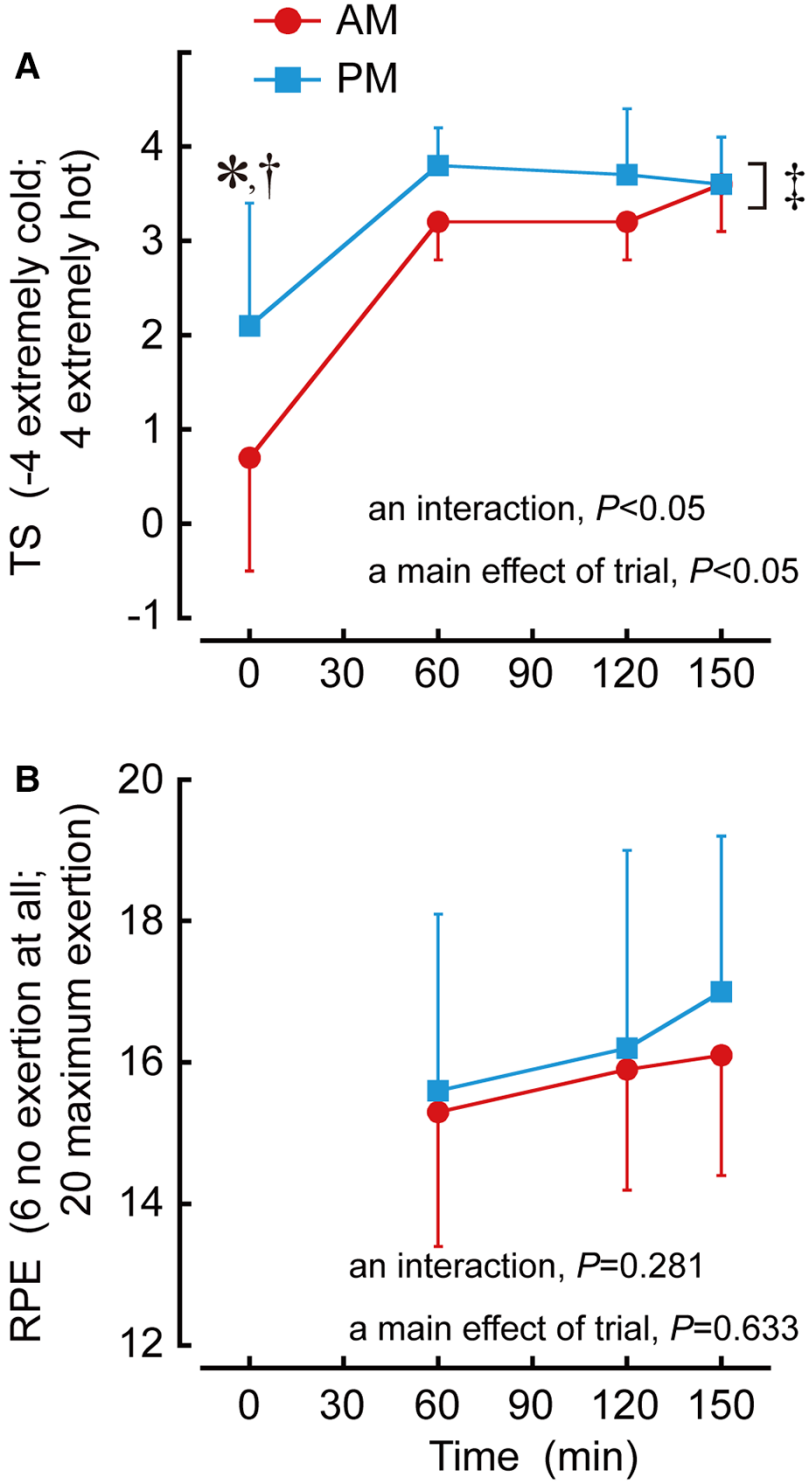
Time-of-day differences in thermal sensation (**A**: TS) and rating of perceived exertion (**B**: RPE) during exercise. *P < 0.05 denotes a difference of pre-exercise between AM and PM trials. †*P *< 0.05 denotes a trial by time interaction between AM and PM trials. ‡*P *< 0.05 denotes a main effect of trial between AM and PM trials.

There was a trial by time interaction in TS (*P *< 0.05; Figure 4A) which was higher in PM than AM trial at 0 min (*P *< 0.01; *d *= 1.12). A main effect of trial during the sessions was apparent for TS (*P *< 0.05) which was higher in PM than AM trial (*P *< 0.05; *d *= 0.51). Without 0 min, there was a main effect of trial in TS (*P *< 0.05) which was higher in PM than AM trial (*P *< 0.05; *d *= 0.97).

There were no trial by time interaction (*P *= 0.633) and main effect of trial (*P *= 0.281) during the sessions in RPE (Figure 4B).

## Discussion

We investigated to what extent the time-of-day affects thermal strain during 2.5-h badminton training sessions in the gym without airflow and air conditioning in hot summer environmental conditions. Pre-exercise T_sk_, T_ty_ and HR were lower in AM than PM trial (Figure 3) which is the common finding in chronobiological studies (2, 3, 24–26). These time-of-day effects would provoke an interaction in T_sk_, T_ty_ and HR which indicate greater increases in these variables during the sessions in AM than PM trial. Moreover, no difference in the average HR during exercise was apparent between AM (75 ± 4% HRmax) and PM (76 ± 5% HRmax) trials. However, given higher T_sk_, T_ty_ and TS and a greater S in PM than AM trial, the present study indicates greater thermal strain during the sessions in PM than AM trial, in contrast to our experimental hypothesis.

In the current study, participants performed exercise in the gym at a high WBGT of 28.1 ± 0.5°C in AM trial and 30.0 ± 0.9°C in PM trial (Figure 2), corresponding to an extreme risk category (≥28°C) for exertional heat-related illness (27). This means that participants in both trials were exposed to greater environmental heat stress. This study shows that indoor environmental heat stress inclines with increasing outdoor environmental heat stress in the morning or declines with decreasing environmental outdoor heat stress in the afternoon (Figure 2). In agreement with the study of Otani et al. (3), these changes led to greater outdoor than indoor environmental heat stress during AM trial and greater indoor than outdoor environmental heat stress during PM trial. This result is in agreement with the common observations of the architectural research concerning the time-of-day effects on the relationships between outdoor and indoor environmental heat stress in a building during the summer heat (28).

The findings of this study are opposite to that of the studies of Otani and colleagues during 3-h baseball (1) and 2-h soccer (2) training sessions in outdoors and 2.5-h judo training sessions in indoors (3) in the summer heat under a clear sky. While no RPE difference between trials in this study was in line with these previous studies, these studies reported higher T_sk_, T_ty_ and HR in AM than PM trial with no difference between trials in TS. These studies therefore concluded greater risks of thermal strain in AM than PM trial owing to an increase in environmental heat stress during AM trial compared with a decrease in environmental heat stress during PM trial. We detected high T_a_ in PM than AM trial (Figure 2) which was consistent with one previous study (3). However, there was high WBGT in PM than AM trial (Figure 2) which was inconsistent with that study (3), albeit there were no T_a_ and WBGT differences between trials in other previous studies (1, 2). These results imply a greater environmental heat stress difference between AM and PM trials in the present study compared with that of the previous studies (1–3).

During heat stress exercise, Hori et al. (29) clearly showed higher T_sk_, T_c_ and HR at 35°C than 30°C T_a_ condition. Moreover, it is well reported higher TS in greater environmental heat stress compared to less environmental heat stress during exercise (30, 31). Lei and colleagues (32) demonstrated that higher T_sk_ and TS during exercise in a climatic chamber at 35°C T_a_ and 50% RH than 29°C T_a_ and 70% RH condition when vapour pressure/absolute humidity was equivalent in both conditions (about 2.8 kPa/ 20 g·m^−3^). In the current study, T_a_ and RH during the sessions averaged 30°C and 74% in AM trial and 33°C and 64% in PM trial when absolute humidity was not different between trials (AM 22.5 g·m^−3^, PM 23.0 g·m^−3^: *P *= 0.315: Figure 2). These results indicate that the humidity effect on thermoregulatory responses may be small between trials (32), although T_a_ was 3°C higher in PM than AM trial. In the heat balance model in the current study (Equation 3 in supplementary material), an individual’s HR, body mass, age, moving velocity and T_sk_ and environmental variables of T_a_, global solar radiation, air pressure, wind speed, floor surface temperature, vapour pressure and relative humidity were used for the calculation. As a result, this 3°C higher T_a_ resulted in less heat loss by radiation (R; Equation 6 in supplementary material) and by convection (C; Equation 15 in supplementary material) in PM than AM trial. These observations suggest that dry (radiation + convection) heat loss was lower in PM than AM trial. These observations would cause a greater S (Equation 3 in supplementary material) in PM than AM trial in conjunction with no difference between trials in M–W (Equatiosn 4, 5 in supplementary material) and E_sk_ (Equation 19 in supplementary material). Considering the observations of these previous studies (29–31) and the results of heat balance model, a 3°C higher T_a_ would elicit higher T_sk_, T_ty_ and TS and a greater S in PM than AM trial. Therefore, greater thermal strain in PM than AM trial may be mainly accompanied by greater environmental heat stress in the gym, involving high T_g_ and WBGT as well as T_a_ in PM than AM trial. Meanwhile, Cramer et al. (33) reported that reduction of skin blood flow by 20% did not alter overall heat loss and this potentially indicates that the overall heat storage could only be affected beyond the skin blood flow threshold. Although the current study did not measure skin blood flow, this might influence the results of overall heat loss response during the sessions.

Another possibility of opposite results between the current and previous studies (1–3) is the absence of airflow. Although there was no airflow in the gym in the present study, previous study settings had natural wind flow of 4.0–13.8 km·h^−1^ in outdoor studies (1, 2) and an artificial airflow of 2.5 km·h^−1^ in the indoor study (3). During heat stress exercise, airflow can elevate EHL and attenuate increases in T_sk_, T_c_ HR and with increasing air velocity (6–9). The absence of airflow in the gym would contribute to low E_sk_ of both trials (Equation 21 in supplementary material) (Table 2) which may highlight a greater S in PM than AM trial in parallel with the results of less heat loss by radiation (R) and by convection (C). Hence, greater environmental heat stress during PM trial may raise thermal strain relative to an increase in environmental heat stress during AM trial when athletes exercise indoors without airflow and air conditioning in the heat. In this study, thus, the absence of airflow in the gym possibly encouraged a rise in thermal strain by the effects of greater environmental heat stress during PM trial rather than an increase in environmental heat stress during AM trial, in contrast to the previous studies (1–3). It is known that airflow must be restricted when playing badminton and table tennis to prevent the wind from affecting the shuttlecock and ball flight (10, 11). Nevertheless, given the beneficial effect of airflow on increasing EHL during exercise-heat stress (6–9), fan use is recommended to facilitate this effect during these training sessions in the heat to the extent possible.

The time-of-day effects of lower pre-exercise T_sk_, T_ty_ and HR resulted in greater increases in these variables during the sessions in AM than PM trial. During exercise-heat stress, these are known that a faster rate of rise in T_sk_ (34), T_c_ (35) and HR (36) causes a reduced endurance performance, owing to an increase in thermoregulatory strain. Also, a faster rate of rise in T_c_ has been shown to induce a reduced muscle force production (37, 38). Hence, exercise performance in AM trial might be low at the later stages of training, while this study did not measure it. Further study is required to examine these influences on exercise performance during the same experiments as this study.

The current study has some important limitations. The present study was restricted to use T_ty_ to estimate T_c_. This is because of difficulty in using rectal temperature for field studies and in using gastrointestinal thermometry for research owing to stringent regulations pertaining to pharmaceutical usage and medical devices in Japan. Previous studies reported that rectal temperature relates to (19) or does not relate to (39) T_ty_ during exercise-heat stress, and therefore the interpretation of T_ty_ data requires caution. However, two studies demonstrated that T_ty_ well correlates with tympanic temperature measured by a contact tympanic thermometer during both lower leg immersion in hot water and cycling exercise in a temperate environment (40) and during cycling exercise in the heat (18). Moreover, AM trial was conducted 22 days after the PM trial date, which may have influenced the training status of the study participants. Therefore, future experiments should employ rectal temperature and conduct the trials in a shorter period to provide a greater validity and reliability in research in terms of preventing exertional heat-related illness in the summer heat.

## Conclusions

We conclude that thermal strain is greater in the late afternoon from 1600 h than morning from 0900 h in high school athletes during 2.5-h badminton training sessions in the gym without airflow and air conditioning in the summer heat under a clear sky. This is accompanied by higher T_sk_, T_ty_ and TS, and a greater S in PM than AM trial with greater environmental heat stress in the gym in PM trial compared with AM trial. Therefore, athletes and coaches of indoor sports should perceive that athletes may be exposed to a greater risk for thermal strain in the late afternoon from 1600 h than in the morning from 0900 h during the sessions in the gym under these conditions. However, given greater increases in T_sk_, T_ty_ and HR during the sessions in AM than PM trial because of time-of-day effects, athletes should also take care of the risks of thermal strain in the morning from 0900 h in such circumstnces.

## Data Availability

The raw data supporting the conclusions of this article will be made available by the authors, without undue reservation.
