# Supplementary material for: Thermal strain is greater in the late afternoon than morning during exercise in the gym without airflow and air conditioning on a clear summer day
Source: Front Sports Act Living. 2023 Feb 28;5:1147845. doi: 10.3389/fspor.2023.1147845 (PMC10011131; doi:10.3389/fspor.2023.1147845)
Supplement: Supplementary file 1 [file Datasheet1.docx]

Supplementary Material

Greater risk of thermal strain in the late afternoon than morning during exercise in the gym without airflow and air conditioning on a clear summer day

Hidenori Otani*, Takayuki Goto, Yuki Kobayashi, Heita Goto, Minayuki Shirato, Yuri Hosokawa, Ken Tokizawa, Mitsuharu Kaya

*** Correspondence:** Hidenori Otani: hotani@himeji-du.ac.jp

**METHODS**

**Calculations**

***Physiological variables***

Mean skin temperature (T_sk_) was calculated as follows (Ramanathan 1964):

(Eq. 1): T_sk_ = 0.3·T_ch_ + 0.3·T_up_ + 0.2·T_th_ + 0.2·T_ca_ [ºC]

where T_ch_ is chest skin temperature, T_up_ is upper arm skin temperature, T_th_ is thigh skin temperature and T_ca_ is calf skin temperature.

Total sweat loss was calculated as follows:

(Eq. 2): Total sweat loss = body mass loss + the volume of water ingested [L].

Age-predicted maximal HR (HRmax) was calculated by subtracting the age from 220.

Heat storage (S) was calculated as the following heat balance model:

(Eq. 3): S = (M – W) ± R ± C ± K − E_sk_ − (C_res_ + E_res_) [W·m^−2^]

where (M – W) is metabolic heat production, M is metabolic rate, W is external work, R is heat exchange by radiation, C is heat exchange by convection, K is heat exchange by conduction, E_sk_ is heat loss by evaporation from the skin, and (C_res_ + E_res_) is respiratory heat loss by convection and evaporation, respectively (all units in W·m^−2^). With R, C and K, positive (+) and negative (−) values are heat gain and loss, respectively. In general, heat exchange by conduction (K) is assumed to be negligible.

Metabolic heat production (M – W) was calculated as M minus W. Metabolic rate (M) was calculated as follows (Keytel et al. 2005):

(Eq. 4): M = [−55.0969 + (0.6309·HR) + (0.1988·body mass) + (0.2017·age)]/4.184·60·T_h_ [W·m^−2^]

where HR is an individual’s average HR during the sessions in bpm (153±8 bpm in AM trial and 155±10 bpm in PM trial), body mass is an individual’s pre-exercise body mass in kg, age is an individual’s age in years and T_h_ is the exercise duration in hour. External work (W) was calculated as follows (ASHRAE 2005):

(Eq. 5): W = µ·M [W·m^−2^]

where µ is the body’s mechanical efficiency (10% in the present study because of assuming 8-10% for running [Nishi 1981]).

In an outdoor environment under the sun, heat exchange by radiation (R) was calculated as follows (Błażejczyk and Matzarakis 2007):

(Eq. 6): R = Q + L [W·m^−2^]

where Q is absorbed short-wave radiation and L is net long-wave radiation (both units in W·m^−2^).

Absorbed short-wave radiation (Q) was calculated as follows (Błażejczyk 2004):

(Eq. 7): Q = (0.0014·K_g_^2^ + 0.476·K_g_ − 3.8)·(1 − 0.01·*ac*)·I_rc_ [W·m^−2^]

where K_g_ is global solar radiation in W·m^−2^, *ac* is albedo of clothing in % (assumed to be 57% for light-coloured clothing [Kenny et al. 2008]) and I_rc_ is coefficient reducing convective and radiative heat transfer through clothing (using the equation below). Coefficient reducing convective and radiative heat transfer through clothing (I_rc_) was calculated as follows (Błażejczyk 2004; Błażejczyk and Matzarakis 2007):

(Eq. 8): I_rc_ = h_cl_/(h_cl_ + h_cr_ + 21.55·10^−8^·T_k_^3^)

where h_cl_ is coefficient of heat transfer through clothing in W·(m^2^·K)^−1^ (using the equation below), h_cr_ is coefficient of convective and radiative heat transfer in W·(m^2^·K)^−1^ (using the equation below) and T_k_ in ambient temperature in K. Coefficient of heat transfer through clothing (h_cl_) was calculated as follows (Błażejczyk 2004; Błażejczyk and Matzarakis 2007):

(Eq. 9): h_cl_ = (0.013·*ap* − 0.04·T_a_ − 0.503)·0.53/{R_cl_·[1 − 0.27·(*v* + *mv*)^0.4^]} [W·(m^2^·K)^−1^]

where *ap* is air pressure in hPa, T_a_ is ambient temperature in °C, R_cl_ is the intrinsic clothing insulation in clo (0.405 in this study, using the equation below), *v* is wind speed in m·s^−1^ and *mv* is an individual’s average moving velocity during the sessions in m·s^−1^ (1.5 in this study; Suda et al. 2004). Coefficient of convective and radiative heat transfer (h_cr_) was calculated as follows (Błażejczyk 2004; Błażejczyk and Matzarakis 2007):

(Eq. 10): h_cr_ = (0.013·*ap* − 0.04·T_a_ − 0.503)·(*v* + *mv*)^0.4^ [W·(m^2^·K)^−1^].

Net long-wave radiation (L) was calculated as follows (Błażejczyk 2004; Błażejczyk and Matzarakis 2007):

(Eq. 11): L = (0.5·L_g_ + 0.5·L_a_ − L_s_)·I_rc_ [W·m^−2^]

where L_g_ is the balance for heat exchange by thermal radiation between human body and the ground in W·m^−2^ (using the equation below) and L_a_ and L_s_ are the balance for heat exchange by thermal radiation between the atmosphere (L_a_) and human body (L_s_) in W·m^−2^ (using the equation below). The balance for heat exchange by thermal radiation between human body and the ground (L_g_) was calculated as follows (Błażejczyk and Matzarakis 2007):

(Eq. 12): L_g_ = ɛ·*σ*·(273+T_gr_)^4^ [W·m^−2^]

where ɛ is the area weighted emissivity coefficient for natural objects (0.97 in the present study), *σ* is the Stefan-Boltzmann constant, 5.67·10^−8^ in W·(m^2^·K^4^)^−1^ and T_gr_ is average floor surface temperature during the sessions in °C (29°C in AM trial and 30°C in PM trial). The balance for heat exchange by thermal radiation between the atmosphere (L_a_) and human body (L_s_) were calculated as follows (Błażejczyk and Matzarakis 2007):

(Eq. 13): L_a_ = ɛ·*σ*·(273 + T_a_)^4^·[0.82 − 0.25·10^(−0.094·^*^vp^*^)^] [W·m^−2^]

where *vp* is vapour pressure in hPa.

(Eq. 14): L_s_ = ɛ_h_·*σ*·(273 + T_sk_)^4^ [W·m^−2^]

where ɛ_h_ is the area weighted emissivity coefficient for human body (0.95 in the present study) and T_sk_ is an individual’s average mean skin temperature during the sessions in °C (34.7±0.6°C in AM trial and 35±0.4°C in PM trial).

Heat exchange by convection (C) was calculated as follows (Błażejczyk and Matzarakis 2007):

(Eq. 15): C = h_cr_·(T_a_ − T_sk_)·I_rc_ [W·m^−2^].

Heat loss by convection and evaporation from respiration (C_res_ + E_res_) was calculated as follows (ASHRAE 2005):

(Eq. 16): C_res_ + E_res_ = 0.0014·M·(34 − T_a_) + 0.0173·M·(5.87 − P_a_) [W·m^−2^]

where P_a_ is the water vapour pressure in the ambient air in kPa (using the equations below). The water vapour pressure in the ambient air (P_a_) was calculated as follows (Parsons 2014):

(Eq. 17): P_a_ = P_sa_·RH [kPa]

where P_sa_ is the saturated water vapour pressure in kPa (using the equations below) and RH is relative humidity. The saturated water vapour pressure (P_sa_) was calculated as follows (Parsons 2014):

(Eq. 18): P_sa_ = 0.1·exp[18.956 − 4030.18/(T + 235)] [kPa]

where T is a temperature in °C.

Heat loss by evaporation from the skin (E_sk_) was calculated as follows (Parsons 2014):

(Eq. 19): E_sk_ = [*w*·(P_sk,s_ − P_a_)]/[R_e,cl_ + (1/*f*_cl_·h_e_)] [W·m^−2^]

where *w* is skin wettedness (assumed to be 0.95 for fully acclimated individuals [Ravanelli et al. 2018]), P_sk,s_ is the partial water vapour pressure at the skin in kPa (assumed to be the saturated water vapour pressure [P_sa_] at T_sk_), R_e,cl_ is the evaporative resistance of clothing in (m^2^·kPa)·W^−1^ (0.01 in both trials), *f*_cl_ is the clothing area factor (1.12 in both trials) and h_e_ is evaporative heat transfer coefficient (using the equations below). Evaporative heat transfer coefficient (h_e_) was calculated as follows (Parsons 2014):

(Eq. 20): h_e_ = 16.5·h_c_ [W·(m^2^·kPa)^−1^]

where h_c_ is convective heat transfer coefficient (using the equations below). Convective heat transfer coefficient (h_c_) was calculated as follows (Parsons 2014):

(Eq. 21): h_c_ = 8.3·*v*^0.6^ [W·(m^2^·kPa)^−1^].

***Environmental variables***

Absolute humidity was calculated as follows (Parsons 2014):

(Eq. 22): Absolute humidity = 2.17·P_a_/T_k_ [kg·m^−3^].

**References**

ASHRAE. (2005). ASHRAE Handbook of Fundamentals. Atlanta: ASHRAE, Inc.

Błażejczyk, K. (2004). Assessment of radiation balance in man in various meteorological and geographical conditions. Geographia Polonica 77, 63-76.

Błażejczyk, K., Matzarakis, A. (2007). Assessment of bioclimatic differentiation of Poland based on the human heat balance. Geographia Polonica 80, 63-82.

Kenny, N.A., Warland, J.S., Brown, R.D., Gillespie, T.G. (2008). Estimating the radiation absorbed by a human. Int J Biometeorol 52, 491-503.

Keytel, L.R., Goedecke, J.H., Noakes, T.D., Hiiloskorpi, H., Laukkanen, R., van der Merwe, L., et al. (2005). Prediction of energy expenditure from heart rate monitoring during submaximal exercise. J Sports Sci. 23, 289-297.

Nishi, Y. (1981). Measurement of thermal balance of man. In: Bioengineering, thermal physiology and comfort. eds. Cena K, Clark JA. Amsterdam: Elsevier. pp. 29-39.

Parsons, K. (2014). Human thermal environments. London: Tayler & Francis.

Ramanathan, N.L. (1964). A new weighting system for mean surface temperature of the human body. J Appl Physiol 19, 531-533.

Ravanelli, N., Coombs, G.B., Imbeault, P., Jay, O. (2018). Maximum Skin Wettedness after Aerobic Training with and without Heat Acclimation. Med Sci Sports Exerc. 50, 299-307.

Suda, K., Umebayashi, K., Sato, Y., Michikami, S. (2004). Automatic measurement of running distance during badminton match using computer-based trace analysis. In: Pre-olympic Congress 2004. Thessaloniki: ICSSPE 2004. p.370.
